# Self-Disclosure and Social Support in a Web-Based Opioid Recovery Community: Machine Learning Analysis

**DOI:** 10.2196/71207

**Published:** 2025-07-17

**Authors:** Yu Chi, Huai-yu Chen, Khushboo Thaker

**Affiliations:** 1 School of Information, College of Information, Data and Society, San Jose State University, 1 Washington Sq, San Jose, CA, 95192, United States, 1 4125396621; 2 Department of Communication, College of Communication and Information, University of Kentucky, Lexington, KY, United States; 3 School of Computing and Information, University of Pittsburgh, Pittsburgh, PA, United States

**Keywords:** substance use, opioid, recovery, Reddit, social media, peer support, online health community, natural language processing

## Abstract

**Background:**

The opioid crisis remains a critical public health challenge, with opioid use disorder (OUD) imposing significant societal and health care burdens. Web-based communities, such as the Reddit community r/OpiatesRecovery, provide an anonymous and accessible platform for individuals in recovery. Despite the increasing use of Reddit for substance use research, limited studies have explored the content and interactions of self-disclosure and social support within these communities.

**Objective:**

This study aims to address the following research questions: (1) What content do users disclose in the community?; (2) What types of social support do users receive?; and (3) How does the content disclosed relate to the type and extent of social support received?

**Methods:**

We analyzed 32,810 posts and 324,224 comments from r/OpiatesRecovery spanning 8 years (2014‐2022) using a mixed method approach. Posts were coded for recovery stages, self-disclosure, and goals, while comments were categorized into informational and emotional support types. A machine learning–based classifier was used to scale the analysis. Regression analyses were conducted to examine the relationship between post content and received support.

**Results:**

The majority of posts were made by individuals using opioids (7225/32,810, 22.0%) or in initial recovery stages (less than 1 mo of abstinence; 27.7%). However, posts by individuals in stable recovery (abstinence for more than 5 years) accounted for only 1.8%. Informational self-disclosure appeared in 88.3% (n=28,977) of posts, while emotional self-disclosure was present in 75.6% (n=24,816). Posts seeking informational support (19,153/32,810, 58.4%) were far more common than those seeking emotional support (779/32,810, 2.4%). On average, each post received 9.88 (SD 11.36) comments. The most frequent types of support were fact and situational appraisal (mean 5.62, SD 6.82) and personal experience (mean 4.88, SD 5.98), while referral was the least common (mean 0.61, SD 0.50). Regression analyses revealed significant relationships between self-disclosure and received support. Posts containing informational self-disclosure were more likely to receive advice (β=0.17, *P*<.001), facts (β=0.30, *P*<.001), and opinions (β=0.11, *P*<.001). Emotional self-disclosure predicted higher levels of emotional support (β=0.17, *P*<.001) and personal experiences (β=0.07, *P*<.001). Posts from individuals in the addiction stage received more advice (β=−0.06, *P*<.001) but less emotional support (β=−0.05, *P*<.001) compared with posts from individuals in later recovery stages.

**Conclusions:**

This study highlights the role of self-disclosure in fostering social support within web-based OUD recovery communities. Findings suggest a need for increasing engagement from individuals in stable recovery stages and improving the diversity and quality of social support. By uncovering interaction patterns, this study provides valuable insights for leveraging online support groups as complementary resources to traditional recovery interventions.

## Introduction

### Background

The opioid crisis remains one of the most pressing public health emergencies globally, with opioid use disorder (OUD) contributing to significant mortality, societal burden, and health care challenges, particularly in the United States [1-3]. Addressing OUD, a chronic and relapsing condition, requires sustained access to effective recovery support systems [4-6]. Peer-based programs like Narcotics Anonymous have been shown to reduce substance use, increase treatment completion, and promote long-term recovery [7]. However, barriers such as stigma, financial difficulties, or logistic issues prevent many from accessing offline, in-person resources [8], highlighting the increasing importance of web-based communities as accessible, anonymous, and scalable recovery support platforms [9,10].

Reddit, a widely used social media platform, hosts numerous topic-focused communities, or “subreddits,” where users can connect, share experiences, and offer support. Among these, r/OpiatesRecovery has emerged as the largest recovery-focused subreddit for individuals with OUD [11]. As of December 2024, it had over 50,000 subscribers, with even nonsubscribed users able to post. With its title line, “You are not alone in this fight,” the subreddit encourages individuals to engage in self-disclosure, the act of sharing personal thoughts, emotions, or experiences [12]. Self-disclosure plays a central role in the recovery process, particularly in digital spaces where anonymity reduces fears of stigma and encourages open dialogue.

Self-disclosure in web-based communities not only facilitates personal expression but also elicits social support from peers who have undergone similar experiences [13,14]. According to social penetration theory (SPT), interpersonal relationships develop through progressive layers of self-disclosure, where deeper and more personal revelations foster greater intimacy and supportive exchange [15]. The relationship between self-disclosure and social support has been examined in other vulnerable or stigmatized contexts in web-based communities. For example, De Choudhury and De [16] found that Reddit users disclosing mental health struggles using emotionally candid (“low inhibition”) language received more social support. Similarly, Andalibi et al [17] revealed that Instagram posts tagged with #depression and disclosed personal narratives, illness, or self-appearance concerns tended to receive more positive social support.

However, in the context of OUD recovery, self-disclosure and social support as well as the relationship between them remain largely underexamined. Existing Reddit-based substance use studies have primarily focused on high-level trending topics and language features [18-20]. A recent review found that only 6 out of 60 studies examined user-to-user interactions, such as seeking and providing support, with even fewer studies focusing specifically on self-disclosure or social support [21]. Moreover, existing studies often rely on small datasets or lack in-depth analysis, restricting their ability to produce generalizable findings applicable to broader OUD recovery communities [22,23].

In recent years, natural language processing (NLP) methods have been widely adopted for analyzing social media data to study health-related web-based communication, particularly at scale [24,25]. In the context of OUD, recent research by Balsamo et al [26] examined peer support within r/OpiatesRecovery, highlighting the importance of recognition, acknowledgment, and knowledge exchange in sustained engagement by analyzing 2125 recovering users. Their work demonstrated the potential of Reddit as a complementary intervention for individuals with OUD through the application of NLP approaches. However, the relationship between the content of self-disclosure and the type of support received is still underexplored.

To address these gaps, we conduct a large-scale, machine learning-driven NLP analysis of r/OpiatesRecovery. Specifically, we analyze 32,810 posts and 324,224 comments spanning 8 years (2014‐2022). Our findings revealed that most posts were made by individuals in addiction or early stages of recovery, while contributions from individuals in long-term recovery were limited. Posts containing informational and emotional self-disclosure were more likely to elicit support from the community, although the type and amount of support varied, underscoring the importance of disclosure in fostering engagement.

This formative research contributes empirical, large-scale evidence to the feasibility of web-based communities like Reddit as peer-driven support systems. The contributions of this study to the field of OUD recovery research are threefold. First, it characterizes the populations using web-based recovery communities, patterns of self-disclosures, and the types of social support they exchange. Second, it contributes empirical evidence to the relationship between self-disclosure and social support for the OUD recovery community. Third, it also discusses design insights for improving web-based recovery communities, such as addressing limited engagement from long-term recovery users and the types of support less prevalent.

### Objectives

This study aims to explore self-disclosure and social support in web-based OUD recovery communities. By leveraging the open-access Reddit data, we seek to answer 3 research questions (RQs): (RQ1) What content do users disclose in a web-based community for opioid recovery?, (RQ2) What types of social support do users receive within the community?, and (RQ3) How does the content disclosed relate to the type and extent of social support received? By addressing these questions, this study enhances our understanding of the role of web-based communities in supporting individuals on their journey of recovery from OUD.

## Methods

### Data Collection

The study used data from r/OpiatesRecovery. Data collection used the Python Reddit Application Programming Interface Wrapper [27] to retrieve all the posts, comments, and associated metadata from January 1, 2014, to May 5, 2022. A line chart of the number of posts and comments from 2014 to 2022 is included in Multimedia Appendix 1.

### Data Preprocessing

Prior to analysis, posts that were deleted or removed at the time of analysis were excluded to ensure the dataset reflected active and accessible content during the study period. In addition, entries with missing or malformed timestamps or text were filtered out. For model training, all text data were tokenized using the WordPiece tokenizer, as implemented by the pretrained BERT-large-uncased model (bidirectional encoder representations from transformers) in the transformers library [28]. While our analysis was conducted at the post and comment level, we also calculated the number of unique users (n=12,375) who contributed posts, to provide additional context on user engagement. However, we did not perform a user-level analysis, as the focus of this study was on the characteristics of individual posts and comments rather than user trajectories. As a result, the dataset consists of 32,810 initial posts and 324,224 corresponding comments.

### Analysis of Initial Posts

Semiopen coding was guided by an initial codebook derived from prior literature and refined through pilot coding. The finalized codebook aimed to identify 5 categories of information: demographic information, informational self-disclosure, emotional self-disclosure [29], opioid use and recovery stages [30,31], and the goal of the post [14]. The descriptions and deidentified examples of each category are provided in Table 1. To ensure reliability, 200 randomly selected posts were independently coded by the first 2 authors, followed by weekly discussions to reconcile any disagreements. This process resulted in a good to a substantial level of agreement across all categories between the 2 annotators, as measured by Cohen κ ranging from 0.55 to 0.78 [32,33]. We then applied the codebook to manually annotate 462 randomly selected posts.

**Table 1.** Codebook for annotating initial posts.

Category, subcategory, and description	Example
Demographic information	
Age, gender, employment if disclosed	“Hey guys. I am a 36 y old man from the US.”
Opioid use and recovery stages	
Addiction: actively using	“i still take codeine.”
Initial recovery: abstinence lasts for less than 1 mo	“Been clean nearly two weeks now…”
Sustained recovery: abstinence lasts for 1 mo to 5 y	“I have 14 mo of sober time from heroin/opiates.”
Stable recovery: abstinence lasts for more than 5 y	“I am an ex-opiate user. abstaining completely for 6 y.”
Unknown stage: cannot tell or not disclosed	—^a^
Informational self-disclosure	
Whether the post contains disclosure of personal information, such as using history, financial information, or trauma experience, etc	“The first time I took hydrocodone I was 13, smoked oxy at 14, injected heroin for the first time at 16.”
Emotional self-disclosure	
Whether the post contains disclosure of personal feelings, such as concerns, frustrations, humiliation, agony, or fears, etc	“I’m terrified of the withdrawal again…”
Goal of the post	
Seeking informational support: whether the post contains the goal of seeking informational support	“What are some methods you guys in recovery use to manage/shape your digestive systems?”
Seeking emotional support: whether the post contains the goal of seeking emotional support	“How long until I turn a corner and feel better? I just need to woman up...pls help me thx.”
Providing informational support: whether the post contains the goal of providing informational support	“The XXX [a medication] did wonders for me made the monster much more bearable. Just wanted to share a different way.”
Providing emotional support: whether the post contains the goal of providing emotional support	“I’m truly blessed and just wanted to share my hope with everyone.”

a No quote provided; the recovery stage could not be inferred from the post.

Given the impracticality of manually annotating all 32,810 posts, we used a machine learning approach by developing BERT-based classifiers for each category using annotated posts, except for demographic information, which was excluded from machine learning due to the limited and sparse data available. We opted for BERT as our foundational NLP model due to its demonstrated superiority in handling the intricate linguistic nuances prevalent in web-based communication, particularly within the specialized context of the r/OpiatesRecovery community. Unlike traditional machine learning models such as term frequency-inverse document frequency or simpler neural networks (eg, long short-term memory networks), which process text sequentially or rely on bag-of-words assumptions, BERT’s bidirectional nature allows it to pretrain on vast amounts of text data, capturing deep contextual relationships between words [28]. This capability is critical for accurately interpreting the often informal, emotionally charged, and jargon-filled language characteristic of web-based recovery forums, where the meaning of a word can be heavily dependent on its surrounding context. Furthermore, while other transformer-based models exist, BERT’s robust pretraining on a large corpus and its proven effectiveness across a wide range of natural language understanding tasks—particularly when fine-tuned for specific classification objectives [34]—made it a well-suited and efficient choice for scaling our analysis from a manually annotated subset to the full dataset of over 32,000 posts. Details of model architecture, training configuration, and hyperparameters are provided in the Multimedia Appendix 2. Our approach maintains interpretability by directly predicting predefined, human-interpretable categories (eg, recovery stage, informational self-disclosure), which allows for intuitive post hoc analysis of community discourse.

Regarding informational and emotional self-disclosure, we exclusively relied on our annotated datasets. To develop the opioid use and recovery stage classifier, we leveraged the Drug Abuse Reddit dataset from Ghosh et al [31], which provided a more balanced distribution of recovery stages. This dataset contains 3151 Reddit posts categorized into 5 recovery stages: “Addicted,” “E-Recovery” (Early), “M-Recovery” (Maintaining), “A-Recovery” (Advanced), and “Others.” We selectively incorporated examples from the “Addicted,” “M-Recovery,” and “A-Recovery” categories to align with our target classification schema: addiction, sustained recovery, and stable recovery. The inclusion of this external data allowed us to address the class imbalance and ensure that the classifier was exposed to sufficient samples from each stage during training, ultimately enhancing the classifier’s performance. In addition, to bolster our annotated dataset for providing informational support and emotional support, we integrated data from prior work, which annotated information and emotional support within 500 comments from a web-based community for individuals with ovarian cancer [35]. Opioid recovery and ovarian cancer represent distinct health contexts, which may influence specific language use and communication norms. However, prior research suggests that core categories of social support, particularly informational and emotional support, are broadly applicable across web-based health communities [36,37]. These support types reflect general communicative functions that transcend disease-specific contexts. This approach enabled us to expand our dataset and improve the robustness of our analysis. Table 2 contains comprehensive information regarding the performance of each model, alongside interrater agreement between human annotators.

**Table 2.** Interrater agreement between human annotators and performance of automatic classifiers for classifying initial posts.

Category	Interrater agreement		Classifiers’ performance		
	Percent agreement	Cohen κ	Precision	Recall	*F*_1_-score
Opioid use and recovery stages	74.3	0.65			
Addiction			0.95	0.83	0.89
Initial recovery			0.64	0.75	0.69
Sustained recovery			0.85	0.93	0.89
Stable recovery			0.91	0.88	0.89
Unknown stage			0.64	0.85	0.73
Informational self-disclosure	96.6	0.78			
Yes			0.94	1.00	0.97
No			1.00	0.67	0.8
Emotional self-disclosure	84.9	0.63			
Yes			0.59	0.68	0.63
No			0.88	0.83	0.85
Providing informational support	91.1	0.60			
Yes			0.88	0.95	0.91
No			0.97	0.92	0.94
Providing emotional support	95.0	0.64			
Yes			0.88	0.93	0.90
No			0.97	0.92	0.94
Seeking informational support	80.4	0.58			
Yes			0.81	0.89	0.85
No			0.93	0.86	0.89
Seeking emotional support	77.7	0.55			
Yes			0.77	0.66	0.71
No			0.77	0.74	0.76

### Analysis of Comments

The analysis of comments focused on identifying 2 types of social support: informational support and emotional support, as these are the most commonly exchanged types of social support in web-based communities [36,37]. Additionally, informational support was further classified into 5 subcategories based on the content of the information: advice, referral, fact and situational appraisal, personal experience, and opinion [38]. The descriptions and corresponding deidentified example comments are provided in Table 3.

To identify support types in comments, we leveraged models trained on annotated data from an ovarian cancer web-based community [35]. Each comment could provide 0, 1, or multiple types of social support. Model details are provided in Multimedia Appendix 2 and an open repository [39]. To assess the models’ transferability to the OUD recovery context, we conducted a manual evaluation on 50 randomly sampled comments per social support category. The first 2 authors, one with expertise in information science and the other in health communication, independently annotated the samples, achieving good to substantial interrater agreement. Discrepancies were resolved through discussion, and model predictions were evaluated against the finalized annotations. All models demonstrated high performance, with *F*_1_-scores ranging from 0.78 to 0.94, indicating reasonable generalizability to the OUD recovery domain. Table 4 shows the interrater agreement between human annotators and model performance across all the social support categories.

**Table 3.** Codebook for annotating social support in comments.

Social support type	Description	Example
Advice	Whether the comment offers ideas and suggestions for coping with challenges	“Find a group/groups and connect with someone who can help. Ask your friends and family (if they know; if not, you might consider telling them, if you think they’d respond in a supportive manner)…”
Referral	Whether the comment refers to information sources, eg, books, websites, contacts.	" Here is one I found very quickly explaining the issue with [a substance] and [a medication] introduction. https://___”
Fact and situational appraisal	Whether the comment offers facts or reassesses the situation.	“For that specific situation, [a medication] implant might be most effective…”
Personal experience	Whether the comment shares personal stories or incidents.	“I am on [a medication] now successfully for at least a year.”
Opinion	Whether the comment offers a view or judgment formed about something. It is not necessarily based on fact or knowledge.	“Also Physical therapy works. It is literally the best thing you can do to manage long term pain”
Emotional support	Whether the comment offers empathy, encouragement, or appreciation.	“Good luck and stay strong”

**Table 4.** Interrater agreement between human annotators and performance of automatic classifiers for classifying social support types in comments.

Category	Interrater agreement		Classifiers’ performance		
	Percent agreement	Cohen κ	Precision	Recall	*F*_1_-score
Advice	96.0	0.91	0.94	0.94	0.94
Referral	98.0	0.90	1.00	0.80	0.89
Fact and situational appraisal	80.0	0.52	0.66	0.95	0.78
Personal experience	94.0	0.88	0.85	0.96	0.90
Opinion	74.0	0.48	0.85	0.88	0.86
Emotional support	96.0	0.92	0.88	0.92	0.90

### Regression Analysis

Pearson correlations were first conducted in our preliminary analysis to identify the relevant variables for inclusion in our model. After confirming adherence to statistical assumptions, we proceeded with multiple regression analysis for each outcome variable, including advice, referral, fact and situational appraisal, personal experience, opinion, and emotional support. As we were interested in comparing the frequency of different types of social support received across various posts, we divided each outcome variable by the total comment count of the respective posts. This normalization process ensures that we can consistently evaluate and compare different types of social support received while mitigating the impact of varying comment counts across posts. Additionally, to facilitate comparisons between each OUD use and recovery stage, we used a dummy coding scheme for the stages, designating the first stage (addiction stage) as our reference group. We selected the addiction group as the reference category, rather than the overall mean, to enable direct comparisons across recovery stages. A sensitivity analysis was conducted to assess potential bias from outliers, and the results showed no significant changes in the findings. Furthermore, a natural log transformation was applied to the referral variable to address the violation of the normality assumption.

### Ethical Considerations

This study was determined to be not human research and received an exemption from review by the Institutional Review Board at the University of Kentucky. The study involved no interaction or intervention with human participants. All data were collected from publicly accessible information on Reddit, and no account or login procedures were required. Data collection procedures adhered to Reddit’s terms of service, ensuring ethical compliance throughout the study. To better protect user privacy, all usernames have been excluded from this paper. In addition, no post titles or URLs are reported, and we avoid quoting content that could reasonably be traced back to specific users.

## Results

### Overview

Among the 462 human-annotated posts, only 5.6% (n=26) of posts disclosed the age of the individual using opioids, which ranged from 14 to 52 years (mean 27.85, SD 7.43). A total of 8.0% (n=37) of posters identified themselves as men (19/462, 4.1%), women (17/462, 3.7%), or nonbinary (1/462, 0.2%). Regarding employment, 16.6% (n=79) of users disclosed their employment status, with 13.6% (n=63) being employed and 6.9% (n=16) unemployed. Due to the low prevalence of demographic self-disclosure, we did not attempt to infer or model the relationship between user demographics and social support patterns. The remaining results presented are based on the full dataset of 32,810 posts and 324,224 comments.

### What Content Do Users Disclose in a Web-Based Community for Opioid Recovery?

**Figure 1.**
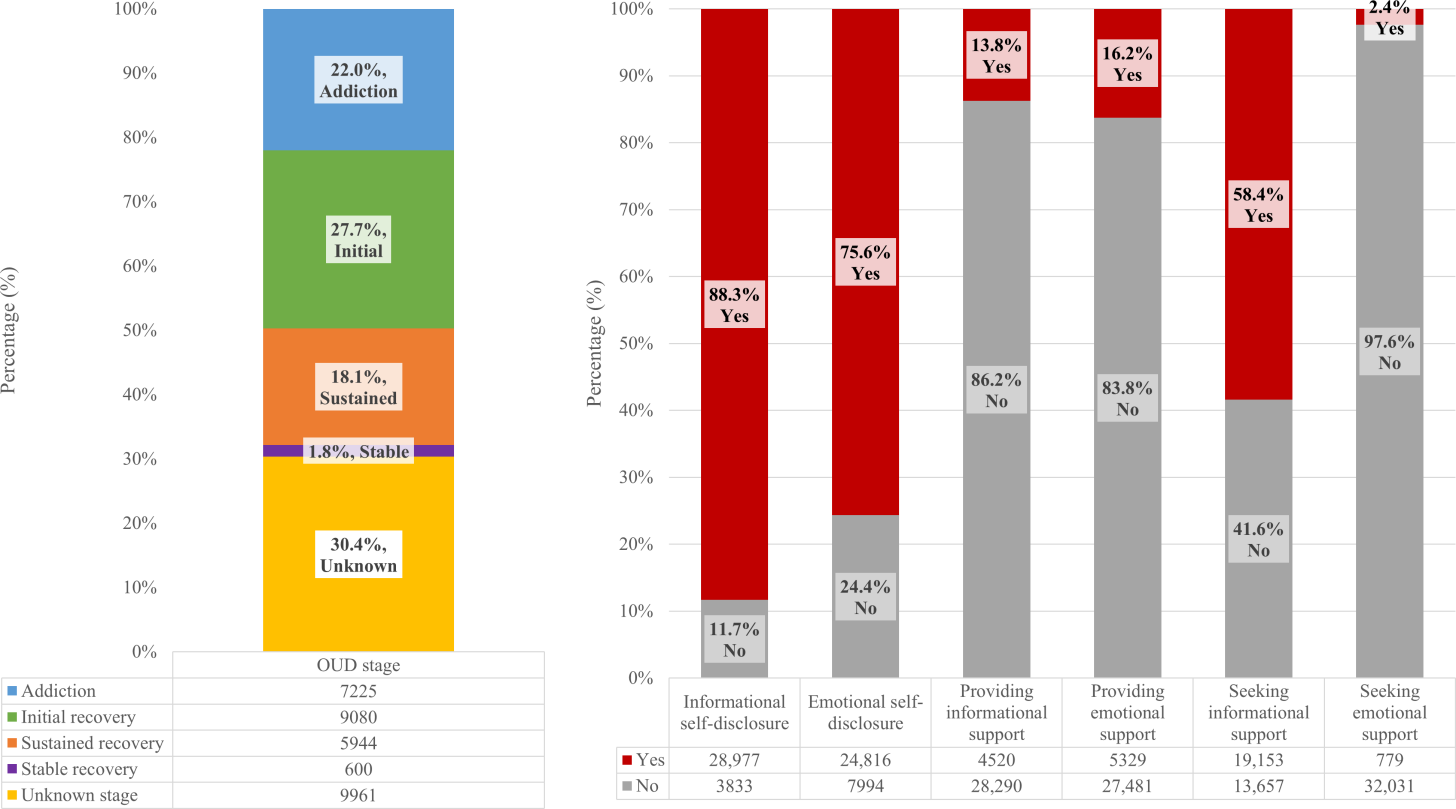
Frequency distribution of posts categorized by content disclosed. OUD: opioid use disorder.

Figure 1 presents the frequency distribution of posts, categorized by the content disclosed within them. Among the 32,810 posts, 22.0% of them (n=7225) were posted by individuals actively using opioids, indicating the addiction stage. Another 27.7% (n=9080) were attributed to the initial recovery stage (abstinence of less than 1 month). Additionally, 18.1% (n=5944) represented the sustained recovery stage (abstinence lasting from 1 month to 5 years), while only 1.8% (n=600) pertained to the stable recovery stage (abstinence exceeding 5 years). Notably, 30.4% (n=9961) of posts did not explicitly disclose their recovery stage. Most posts included informational self-disclosure (28,977/32,810, 88.3%) and emotional self-disclosure (24,816/32,810, 75.6%). However, most posts did not provide informational support (28,290/32,810, 86.2%) or emotional support (27,481/32,810, 83.8%). It is noteworthy that a higher percentage of posts sought informational support (19,153/32,810, 58.4%), while only a few posts explicitly sought emotional support (779/32,810, 2.4%).

### What Types of Social Support Do Users Receive Within the Community?

Table 5 presents the average number of comments per post, the distribution of different types of social support embedded within those comments, as well as correlations among the various types of social support. On average, each post receives approximately 9.88 (SD 11.36) comments. Fact and situational appraisal emerged as the most common form of support, with an average of 5.62 (SD 6.82) comments per post. This was followed by the sharing of personal experiences, averaging 4.88 (SD 5.98) comments per post. Opinions, which reflect individual viewpoints, accounted for an average of 4.36 (SD 5.60) comments per post. Emotional support constituted an average of 3.85 (SD 4.81) comments, while advice was present in approximately 2.61 (SD 3.45) comments on average. Notably, referrals appear less frequently, with posts typically containing fewer than one comment providing referral details, averaging 0.61 (SD 0.50) comments. Table 5 also depicts Pearson correlations among the number of comments and different types of social support. Positively significant correlations were observed for all pairs of variables.

**Table 5.** Pearson correlations among the number of comments and the number of different types of social support.

	Mean (SD)	Comment number	Advice	Referral	Fact and situational appraisal	Personal experience	Opinion	Emotional support
Comment number	9.88 (11.36)	^—a^	0.725^b^	0.357^b^	0.893^b^	0.918^b^	0.891^b^	0.837^b^
Advice	2.61 (3.45)	0.725^b^	—	0.267^b^	0.845^b^	0.722^b^	0.796^b^	0.639^b^
Referral	0.16 (0.50)	0.357^b^	0.267^b^	—	0.323^b^	0.313^b^	0.335^b^	0.266^b^
Fact and situational appraisal	5.62 (6.82)	0.893^b^	0.845^b^	0.323^b^	—	0.910^b^	0.927^b^	0.692^b^
Personal experience	4.88 (5.98)	0.918^b^	0.722^b^	0.313^b^	0.910^b^	—	0.903^b^	0.803^b^
Opinion	4.36 (5.60)	0.891^b^	0.796^b^	0.335^b^	0.927^b^	0.903^b^	—	0.770^b^
Emotional support	3.85 (4.81)	0.837^b^	0.639^b^	0.266^b^	0.692^b^	0.803^b^	0.770^b^	—

a Not applicable.

b All the correlations are significant at *P*<.001.

### How Does the Content Disclosed Relate to the Type and Extent of Social Support Received?

Results from multiple regression analysis for the number of comments with advice revealed that all predictors together explained 11.9% of the variance in the number of comments with advice (*R^2^*=0.12, *F*_10, 30,470_=412.06, *P*<.001). For the number of referrals, the regression analysis showed that stable recovery, informational self-disclosure, emotional self-disclosure, providing informational support, providing emotional support, and seeking informational support collectively accounted for 3.0% of the variance (*R^2^*=0.03, *F*_10, 3971_=12.16, *P*<.001). Similarly, the analysis for fact and situational appraisal indicated that initial recovery, sustained recovery, stable recovery, unknown stage, informational self-disclosure, emotional self-disclosure, providing informational support, providing emotional support, and seeking informational support jointly explained 18.7% of the variance (*R^2^*=0.19, *F*_10, 30,470_=699.48, *P*<.001). The analysis further revealed that stable recovery, emotional self-disclosure, providing informational support, providing emotional support, and seeking informational support together explained 3.0% of the variance in the number of personal experiences (*R^2^*=0.03, *F*_10, 30,470_=93.32, *P*<.001). Regarding the number of opinions, the regression analysis demonstrated that initial recovery, sustained recovery, informational self-disclosure, emotional self-disclosure, providing emotional support, seeking informational support, and seeking emotional support jointly accounted for 4.8% of the variance (*R^2^*=0.05, *F*_10, 30,470_=153.59, *P*<.001). Last, the analysis for the number of comments with emotional support indicated that all the predictors except for informational self-disclosure collectively explained 13.2% of the variance (*R^2^*=0.13, *F*_10, 30,470_=463.18, *P*<.001). Table 6 contains a summary of the regression. A detailed statistical report is in Multimedia Appendix 1.

**Table 6.** Summary of hierarchical regression analysis for variables predicting satisfaction.

Variables	Advice	Referral^a^	Fact and situational appraisal	Personal experience	Opinion	Emotional support
Initial recovery^b^						
β	−0.06	−0.03	−0.06	0.01	−0.11	0.03
*P* value	<.001	.21	<.001	.35	<.001	<.001
Sustained recovery^b^						
β	−0.08	−0.01	−0.10	−0.01	−0.03	0.08
*P* value	<.001	.74	<.001	.11	<.001	<.001
Stable recovery^b^						
β	−0.05	0.04	−0.05	−0.01	−0.01	0.02
*P* value	<.001	.01	<.001	.04	.21	<.01
Unknown stage^b^						
β	−0.10	0.02	−0.07	−0.02	0.00	−0.05
*P* value	<.001	.29	<.001	.06	.70	<.001
Informational self-disclosure						
β	0.02	0.11	0.04	0.00	0.02	−0.01
*P* value	<.01	<.001	<.001	.73	<.01	.38
Emotional self-disclosure						
β	0.12	−0.08	0.05	0.07	0.14	0.17
*P* value	<.001	<.001	<.001	<.001	<.001	<.001
Providing informational support						
β	−0.08	−0.10	−0.07	0.04	−0.01	0.03
*P* value	<.001	<.001	<.001	<.001	.39	<.001
Providing emotional support						
β	−0.09	0.09	−0.13	−0.10	−0.07	0.10
*P* value	<.001	<.001	<.001	<.001	<.001	<.001
Seeking informational support						
β	0.17	0.09	0.30	0.12	0.11	−0.23
*P* value	<.001	<.001	<.001	<.001	<.001	<.001
Seeking emotional support						
β	0.03	0.01	0.01	0.00	0.01	0.05
*P* value	<.001	.60	.23	.62	.02	<.001
Adjusted *R^2^*	0.12	0.03	0.19	0.03	0.05	0.13
*R^2^*	0.12	0.03	0.19	0.03	0.05	0.13

a Natural log transformation was applied to address the violation of the normality assumption.

b The initial recovery, sustained recovery, stable recovery, and unknown stage were dummy-coded to compare with the addition stage.

## Discussion

### Content Disclosed in the Web-Based Opioid Recovery Community

Even though results based on the randomly sampled 462 posts revealed that the majority of posters did not disclose personal details such as age, gender, or employment status, other aspects of informational self-disclosure, such as financial status and history, were prevalent across all the analyzed posts (28,977/32,810, 88.3%). Emotional self-disclosure was also markedly present (24,816/32,810, 75.6%). The finding aligns with the inherent anonymity of web-based platforms, which often encourage personal self-disclosure [40]. Notably, an insubstantial fraction of posts (779/32,810, 2.4%) conveyed a quest for emotional support, while more than half (19,153/32,810, 58.4%) aimed at seeking informational support from the community. These patterns suggest a preference for seeking information—such as ideas, insights, or advice—within the community, rather than seeking emotional or moral solidarity. However, the results raise questions about whether web-based communities like Reddit are a good resource for OUD-related advice. Are there sufficient experienced and knowledgeable members providing informational support in the community?

Our findings regarding the trajectory of the recovery stage further underscore this concern. Notably, the vast majority of posts were centered on initial recovery (27.7%, abstinence lasts for less than 1 mo) or addiction (22.0%, actively using). In contrast, posts from individuals in stable recovery, those who have sustained abstinence for over 5 years, accounted for only 1.8% of all posts. This imbalance highlights the pressing need for support, especially in the vulnerable initial and early stages of recovery. These stages are often marked by uncertainty, intense challenges, and the need for consistent guidance [41,42]. However, the scarcity of contributions from individuals in long-term recovery, those who have successfully navigated the extended journey of recovery, suggested a potential lack of experienced guidance within the r/OpiatesRecovery subreddit.

Several strategies can be devised to bridge the gap and enrich the community’s collective experience and knowledge base. First, dedicated threads for sharing success stories could be created and pinned to encourage long-term users to share their experiences and continue their engagement [43]. Reddit’s social norms inherently promote mutual support and experience sharing [44]. In addition, health advocates are encouraged to use Reddit as a valuable channel for disseminating educational resources, articles, and interventions. For example, Clendennen et al [45] conducted a longitudinal investigation involving 5482 students, revealing a positive relationship between exposure to social media tobacco messages and self-reported tobacco use. Their findings suggest that exposure to health-related messages, including posts on Reddit, might shape social media users’ health behavior. In light of these findings, public health agents can play a pivotal role in enhancing Redditors’ awareness of accessible resources and potentially influencing their health-related behaviors.

### Social Support in the Web-Based Opioid Recovery Community

Regarding the amount of each type of social support, a large number of fact and situational appraisals were first noticed. This might indicate a collective understanding that recovery is not just an emotional journey, but also one requiring concrete steps, knowledge, and understanding [46,47]. The sharing of facts and knowledge about OUD and recovery can potentially empower peer users in the community, providing them with tangible guidance they can apply in their own recovery journey. However, particular caution should be paid to the quality and accuracy of the information, including facts and advice, shared in web-based communities like Reddit. For example, ElSherief et al [48] discovered that the myth that medication treatment for OUD, such as agonist therapy or medication-assisted treatment, merely substitutes one drug for another is present in 4 out of 1000 posts on web-based health communities like Reddit and Drugs-Forum. Users should be encouraged to cross-reference the information they acquire with credible sources and consult professionals to ensure informed decisions in their recovery.

Furthermore, the prevalence of personal experiences underscores the value of storytelling in recovery. Efforts should be made to enhance and promote storytelling among individuals dealing with OUD. This could involve implementing digital storytelling tools, organizing workshops, and providing resources that empower individuals to share their experiences and journeys toward recovery [49,50]. In addition, the limited number of referrals provided is noteworthy. Similar results were revealed by D’Agostino et al [22]. Through analyzing 100 posts related to OUD on Reddit, they found that only 15% of the posts direct individuals with OUD issues to health care professionals. Resources shared by peers in web-based communities could potentially promote resource dissemination and benefit members [51]. However, the limited sharing of resources among individuals with OUD raises questions: Is it due to the lack of user-preferred credible resources for individuals with OUD or the lack of awareness of available resources? Further exploration is required to understand the reasons behind the limited sharing of resources among users with OUD.

### Relationship Between Disclosure and Received Social Support

The multiple regression results offer a nuanced understanding of how post content interacts with the types of social support provided within comments in the web-based community. We first found a differential pattern of support across the recovery stages. Specifically, posts indicating a current addiction stage tend to receive significantly more advice and facts from the community, but less emotional support compared with posts that reveal other stages of recovery. This pattern may reflect the community’s focus on offering tangible solutions to individuals actively struggling with addiction. However, the reduced emotional support for this group suggests a potential area of improvement, as individuals in the addiction might also benefit from empathetic interactions [52].

The findings also emphasize the critical role of self-disclosure in shaping the dynamics of community interactions. Our results indicate that posts containing self-disclosures generally receive more comments offering social support than those without such disclosures. Such observations align with recent Reddit-based research in the context of female infertility [53]. This pattern is also consistent with SPT [15], which posits that individuals tend to respond to others’ disclosures based on the norm of reciprocity. That is, as one person reveals personal information, others feel encouraged to reciprocate with similarly meaningful responses [54]. In the context of r/OpiatesRecovery, self-disclosure functions as a relational cue, signaling openness and vulnerability that invite deeper engagement from community members.

More importantly, the type of self-disclosure affects the type and variety of social support received. Our results indicated emotional self-disclosure posts prompt more diverse and affectively rich responses (eg, emotional support and personal experience) than those focused solely on factual or situational information. This finding aligns with SPT, which suggests that emotional self-disclosures represent a deeper and more intimate layer of self-revelation, which tends to foster stronger interpersonal resonance and encourage others to respond with similarly deep responses [55]. Previous experimental research in both web-based and in-person contexts has also shown that emotional content promotes reciprocal self-disclosure, encouraging others to respond more openly and supportively [56].

Interestingly, posts with emotional self-disclosure tend to gather fewer referrals than those without emotional self-disclosure. This may reflect the nature of emotional disclosures as relational rather than instrumental cues, as suggested by SPT, which may lower the salience of problem-solving responses like referrals and increase the likelihood of affective support[55]. When Reddit users share emotionally vulnerable content, it may evoke a sense of sympathy, closeness, or relational depth among community members. As a result, responders might prioritize empathy and subjective perspectives over pointing to external resources.

It is also important to consider how Reddit differs from other social media platforms in supporting individuals with OUD. Reddit’s affordances (eg, anonymous, long-form posting, and topic-specific subreddits) may foster an environment where users often feel more comfortable engaging in candid self-disclosure and sustained peer-to-peer support. In contrast, Twitter is a public platform where visibility is shaped by algorithms and brevity is prioritized. Prior research indicates that Twitter tends to be institution-driven, with a significant presence in addiction treatment marketing and professional accounts. For example, Russell et al [57] found that 27.4% of opioid-related tweets were associated with addiction treatment marketing, some of which promoted scientifically unsupported claims. Similarly, Tofighi et al [58] reported that 24.6% of opioid-related tweets came from private residential or detoxification programs, while only 4.7% were authored by people who use opioids. In our Reddit dataset (462 human-annotated posts), none were authored by institutions. These findings suggest that Twitter serves both as a platform for recovery support and as a channel for institutional messaging and commercial promotion—unlike Reddit, which fosters greater narrative depth and peer engagement among individuals with lived experience.

Facebook, though more community-oriented, differs in that it typically requires some form of identity verification to join private groups. This partial transparency may encourage more Facebook users to offer social support, as the perceived accountability can foster a stronger sense of community than the fully anonymous environment on Reddit. For instance, Ellway et al [59] analyzed over 14,000 posts from the Australian Facebook page “Never Give Up Giving Up Ice, Drugs” and found that 24.2% of these posts provided informational support while 42.1% offered emotional support. In contrast, our findings from Reddit showed lower rates of support provided, with 13.5% of posts offering informational support and 16.3% providing emotional support. These distinctions highlight how different platform designs shape the nature of recovery dialogue and support. To conclude, Reddit’s anonymity may invite more vulnerable disclosures, while Facebook’s identity-linked structure may encourage more expert-driven support. Twitter, by contrast, appears better suited for rapid information dissemination, public awareness-raising, and promotional messaging. Future research should consider triangulated cross-platform comparisons to better understand how structural and functional differences across social media platforms influence recovery experiences and outcomes.

### Limitations and Future Directions

This study has several limitations. First, our data was exclusively collected from the r/OpiatesRecovery subreddit, which introduces potential sampling bias. The dataset represents a distinct subset of individuals in opioid recovery who actively engage with Reddit communities, potentially differing from those who do not participate in such web-based communities. Therefore, the generalizability of our findings beyond the Reddit community is constrained. Caution should be noted when generalizing these insights to the broader population of individuals in opioid recovery. Moreover, the data gathered from open Reddit discussions is self-reported and lacks clinical verification, limiting the reliability of our dataset [21]. Second, there is a scarcity of demographic information, such as age, gender, and location information, as well as long-term opioid use and recovery. While we manually coded a small set of posts for basic demographic information, only a small percentage of users disclosed such information, which limited our ability to analyze how support patterns may vary by demographics. Access to such demographic and long-term recovery data could provide deeper insights into the opioid recovery landscape and enhance future studies. Third, while we conducted a manual evaluation to confirm the applicability of support-type classification models trained on non-OUD forums, we acknowledge the risk of domain shift. The language used in OUD communities may include slang or emotionally intense narratives that differ from those in cancer-related forums. As such, the models may underperform in capturing OUD-specific linguistic nuances. Future work should consider domain-adaptive training or fine-tuning approaches to enhance model validity within substance use recovery contexts. Fourth, another limitation concerns the detection of emotional content. While the informational self-disclosure classifier performed well (*F*_1_=0.97), the emotional self-disclosure classifier showed lower performance (*F*_1_=0.63), potentially leading to the underrepresentation of emotional content in our analyses. Similarly, the low rate of posts classified as seeking emotional support (2.4%) may reflect both actual user behavior and the model’s difficulty detecting subtle or indirect emotional expressions. For example, a post describing feelings of isolation might implicitly seek empathy and connection, even if it is framed as a request for advice on finding local support groups. Such cases may lead to an underestimation of the true prevalence of emotional support-seeking in the dataset. Future work should explore improved emotional modeling, such as fine-tuned lexicons, affective embeddings, or human-in-the-loop methods to better capture emotional nuance in web-based recovery discussions [60,61]. Last, we acknowledge that deleted Reddit posts may lead to an incomplete representation of self-disclosure and support patterns, particularly for vulnerable or stigmatized groups, which may potentially impact our findings. These limitations should be noted when interpreting our findings and highlight the need for future research to provide a more comprehensive understanding of the complex dynamics within web-based communities focusing on opioid recovery.

### Conclusions

In conclusion, this study used a machine-learning approach to analyze a large-scale dataset, focusing on self-disclosure and social support interactions within a web-based opioid recovery community. The findings revealed that most posts were made by individuals in early recovery stages, with informational and emotional self-disclosure being prevalent and often eliciting more support. Most posts seek informational support rather than emotional support. Fact and situational appraisal was the most common type of social support provided. These results offer valuable insights into the recovery experiences and peer interactions of individuals with OUD in web-based communities. While highlighting the potential of these platforms to complement traditional recovery interventions, the study also identifies areas for improvement, such as fostering long-term engagement and enhancing the quality of shared information. Future research should address these gaps to maximize the effectiveness of online support groups.

## Supplementary material

10.2196/71207 Multimedia Appendix 1 Supplementary results.

10.2196/71207 Multimedia Appendix 2 Model configuration and hyperparameters.

## Abbreviations

BERT: Bidirectional Encoder Representations from Transformers
NLP: natural language processing
OUD: opioid use disorder
RQ: research question
SPT: social penetration theory
