# Supplementary material for: Self-Disclosure and Social Support in a Web-Based Opioid Recovery Community: Machine Learning Analysis
Source: JMIR Form Res. 2025 Jul 17;9:e71207. doi: 10.2196/71207 (PMC12289226; doi:10.2196/71207)
Supplement: Multimedia Appendix 1 [file formative-v9-e71207-s001.docx]

**Appendix 1. Supplementary Results**

**Appendix 1. Figure S1.** Trends in post and comment numbers (2014–2022).


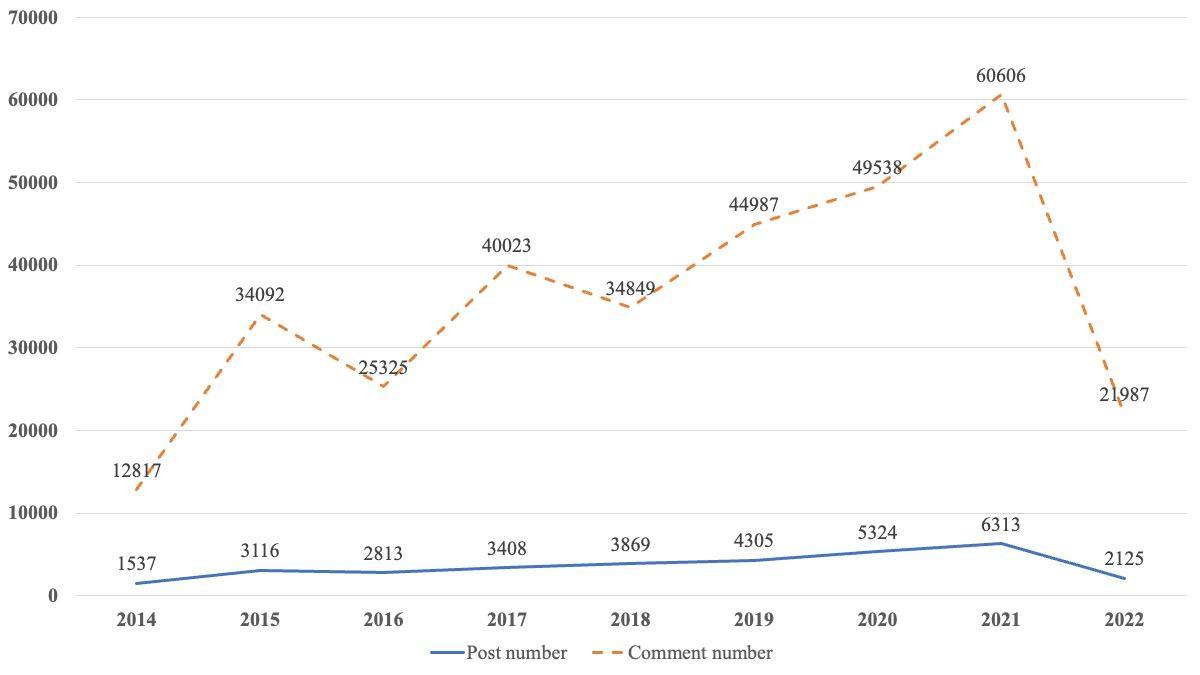


Figure S1 presents the trends in post and comment numbers spanning from January 1, 2014, to May 5, 2022. It suggests a growth within the community over the years, peaking in 2021. Comment numbers exhibited steady growth from 2018 to 2021, with a notable surge during the COVID-19 pandemic (2020-2021). Notably, the decrease observed in 2022 is likely due to the timing of data collection (May 2022).

**Appendix 1. Table S1**

Summary of hierarchical regression analysis on advice.

| **Variables** | | **Step 1** | **Step 2** | | **Step 3** | **Step 4** |
| --- | --- | --- | --- | --- | --- | --- |
| **Recovery stage** | |  |  | |  |  |
|  | Initial recovery | -0.08*^a^* | -0.08*^a^* | -0.08*^a^* | | -0.06*^a^* |
|  | Sustained recovery | -0.13*^a^* | -0.13*^a^* | -0.11*^a^* | | -0.08*^a^* |
|  | Stable recovery | -0.08*^a^* | -0.08*^a^* | -0.06*^a^* | | -0.05*^a^* |
|  | Unknown stage | -0.24*^a^* | -0.15*^a^* | -0.1*^a^* | | -0.10*^a^* |
| **Type of self-disclosure** | |  |  | |  |  |
|  | Informational self-disclosure |  | 0.10*^a^* | 0.02*^b^* | | 0.02*^b^* |
|  | Emotional self-disclosure |  | 0.11*^a^* | 0.10*^a^* | | 0.12*^a^* |
| **Goal of post** | |  |  | |  |  |
|  | Providing informational support |  |  | -0.12*^a^* | | -0.08*^a^* |
|  | Providing emotional support |  |  | -0.12*^a^* | | -0.09*^a^* |
|  | Seeking informational support |  |  |  | | 0.17*^a^* |
|  | Seeking emotional support |  |  |  | | 0.03*^a^* |
| *Adj. R^2^* | | 0.04 | 0.07 | | 0.09 | 0.12 |
| *R^2^* | | 0.04 | 0.07 | | 0.09 | 0.12 |
| *ΔR^2^* | | 0.04*^a^* | 0.03*^a^* | | 0.02*^a^* | 0.03*^a^* |

*Note. All regression coefficients were standardized.*

*^a^ The association is significant at a significance level of .001 (two-tailed).*

*^b^ The association is significant at a significance level of .01 (two-tailed).*

**Appendix 1. Table S2**

Summary of hierarchical regression analysis on referral.

| **Variables** | | **Step 1** | **Step 2** | | **Step 3** | **Step 4** |
| --- | --- | --- | --- | --- | --- | --- |
| **Recovery stage** | |  |  | |  |  |
|  | Initial recovery | -0.02 | -0.03 | -0.03 | | -0.03 |
|  | Sustained recovery | -0.01 | -0.01 | -0.01 | | -0.01 |
|  | Stable recovery | 0.04^c^ | 0.04^c^ | 0.04^c^ | | 0.04^b^ |
|  | Unknown stage | -0.02 | 0.01 | 0.03 | | 0.02 |
| **Type of self-disclosure** | |  |  | |  |  |
|  | Informational self-disclosure |  | 0.13^a^ | 0.10^a^ | | 0.11^a^ |
|  | Emotional self-disclosure |  | -0.07^a^ | -0.09^a^ | | -0.08^a^ |
| **Goal of post** | |  |  | |  |  |
|  | Providing informational support |  |  | -0.12^a^ | | -0.1^a^ |
|  | Providing emotional support |  |  | 0.08^a^ | | 0.09^a^ |
|  | Seeking informational support |  |  |  | | 0.09^a^ |
|  | Seeking emotional support |  |  |  | | 0.01 |
| *Adj. R^2^* | | 0.00 | 0.00 | | 0.01 | 0.02 |
| *R^2^* | | 0.00 | 0.00 | | 0.01 | 0.02 |
| *ΔR^2^* | | 0.00 | 0.00 | | 0.01^a^ | 0.01^a^ |

*Note. All regression coefficients were standardized.*

*^a^ The association is significant at a significance level of .001 (two-tailed).*

*^b^ The association is significant at a significance level of .01 (two-tailed).*

*^c^ The association is significant at a significance level of .05 (two-tailed).*

**Appendix 1. Table S3**

Summary of hierarchical regression analysis on fact and situational appraisal.

| **Variables** | | **Step 1** | **Step 2** | | **Step 3** | **Step 4** |
| --- | --- | --- | --- | --- | --- | --- |
| **Recovery stage** | |  |  | |  |  |
|  | Initial recovery | -0.09^a^ | -0.09^a^ | -0.08^a^ | | -0.06^a^ |
|  | Sustained recovery | -0.18^a^ | -0.18^a^ | -0.15^a^ | | -0.10^a^ |
|  | Stable recovery | -0.10^a^ | -0.10^a^ | -0.07^a^ | | -0.05^a^ |
|  | Unknown stage | -0.20^a^ | -0.13^a^ | -0.07^a^ | | -0.07^a^ |
| **Type of self-disclosure** | |  |  | |  |  |
|  | Informational self-disclosure |  | 0.14^a^ | 0.03^a^ | | 0.04^a^ |
|  | Emotional self-disclosure |  | 0.02^b^ | 0.01 | | 0.05^a^ |
| **Goal of post** | |  |  | |  |  |
|  | Providing informational support |  |  | -0.15^a^ | | -0.07^a^ |
|  | Providing emotional support |  |  | -0.17^a^ | | -0.13^a^ |
|  | Seeking informational support |  |  |  | | 0.30^a^ |
|  | Seeking emotional support |  |  |  | | 0.01 |
| *Adj. R^2^* | | 0.00 | 0.04 | | 0.06 | 0.11 |
| *R^2^* | | 0.00 | 0.04 | | 0.06 | 0.11 |
| *ΔR^2^* | | 0.00 | 0.04^a^ | | 0.02^a^ | 0.05^a^ |

*Note. All regression coefficients were standardized.*

*^a^ The association is significant at a significance level of .001 (two-tailed).*

*^b^ The association is significant at a significance level of .01 (two-tailed).*

**Appendix 1. Table S4**

Summary of hierarchical regression analysis on personal experience.

| **Variables** | | **Step 1** | **Step 2** | | **Step 3** | **Step 4** |
| --- | --- | --- | --- | --- | --- | --- |
| **Recovery stage** | |  |  | |  |  |
|  | Initial recovery | -0.01 | -0.01 | 0.00 | | 0.01 |
|  | Sustained recovery | -0.05^a^ | -0.05^a^ | -0.03^a^ | | -0.01 |
|  | Stable recovery | -0.03^a^ | -0.03^a^ | -0.02^b^ | | -0.01^c^ |
|  | Unknown stage | -0.06^a^ | -0.03^a^ | -0.02 | | -0.02 |
| **Type of self-disclosure** | |  |  | |  |  |
|  | Informational self-disclosure |  | 0.03^a^ | 0.00 | | 0.00 |
|  | Emotional self-disclosure |  | 0.05^a^ | 0.06^a^ | | 0.07^a^ |
| **Goal of post** | |  |  | |  |  |
|  | Providing informational support |  |  | 0.02^c^ | | 0.04^a^ |
|  | Providing emotional support |  |  | -0.11^a^ | | -0.10^a^ |
|  | Seeking informational support |  |  |  | | 0.12^a^ |
|  | Seeking emotional support |  |  |  | | 0.00 |
| *Adj. R^2^* | | 0.00 | 0.00 | | 0.01 | 0.02 |
| *R^2^* | | 0.00 | 0.00 | | 0.01 | 0.02 |
| *ΔR^2^* | | 0.00 | 0.00^a^ | | 0.00^a^ | 0.01^a^ |

*Note. All regression coefficients were standardized.*

*^a^ The association is significant at a significance level of .001 (two-tailed).*

*^b^ The association is significant at a significance level of .01 (two-tailed).*

*^c^ The association is significant at a significance level of .05 (two-tailed).*

**Appendix 1. Table S5**

Summary of hierarchical regression analysis on opinion.

| **Variables** | | **Step 1** | **Step 2** | | **Step 3** | **Step 4** |
| --- | --- | --- | --- | --- | --- | --- |
| **Recovery stage** | |  |  | |  |  |
|  | Initial recovery | -0.12^a^ | -0.12^a^ | -0.11^a^ | | -0.11^a^ |
|  | Sustained recovery | -0.06^a^ | -0.06^a^ | -0.05^a^ | | -0.03^a^ |
|  | Stable recovery | -0.03^a^ | -0.03^a^ | -0.01^c^ | | -0.01 |
|  | Unknown stage | -0.10^a^ | -0.02^b^ | 0.00 | | 0.00 |
| **Type of self-disclosure** | |  |  | |  |  |
|  | Informational self-disclosure |  | 0.06^a^ | 0.02^b^ | | 0.02^b^ |
|  | Emotional self-disclosure |  | 0.12^a^ | 0.12^a^ | | 0.14^a^ |
| **Goal of post** | |  |  | |  |  |
|  | Providing informational support |  |  | -0.03^a^ | | -0.01 |
|  | Providing emotional support |  |  | -0.08^a^ | | -0.07^a^ |
|  | Seeking informational support |  |  |  | | 0.11^a^ |
|  | Seeking emotional support |  |  |  | | 0.01^c^ |
| *Adj. R^2^* | | 0.00 | 0.01 | | 0.03 | 0.04 |
| *R^2^* | | 0.00 | 0.01 | | 0.03 | 0.04 |
| *ΔR^2^* | | 0.00 | 0.01^a^ | | 0.02^a^ | 0.01^a^ |

*Note. All regression coefficients were standardized.*

*^a^ The association is significant at a significance level of .001 (two-tailed).*

*^b^ The association is significant at a significance level of .01 (two-tailed).*

*^c^ The association is significant at a significance level of .05 (two-tailed).*

**Appendix 1. Table S6**

Summary of hierarchical regression analysis on emotional support.

| **Variables** | | **Step 1** | **Step 2** | | **Step 3** | **Step 4** |
| --- | --- | --- | --- | --- | --- | --- |
| **Recovery stage** | |  |  | |  |  |
|  | Initial recovery | 0.05^a^ | 0.05^a^ | 0.05^a^ | | 0.03^a^ |
|  | Sustained recovery | 0.15^a^ | 0.14^a^ | 0.12^a^ | | 0.08^b^ |
|  | Stable recovery | 0.05^a^ | 0.05^a^ | 0.03^a^ | | 0.02^b^ |
|  | Unknown stage | -0.07^a^ | -0.01 | -0.05^a^ | | -0.05^a^ |
| **Type of self-disclosure** | |  |  | |  |  |
|  | Informational self-disclosure |  | -0.08^a^ | 0.00 | | -0.01 |
|  | Emotional self-disclosure |  | 0.20 | 0.20^a^ | | 0.17^a^ |
| **Goal of post** | |  |  | |  |  |
|  | Providing informational support |  |  | 0.08^a^ | | 0.03^a^ |
|  | Providing emotional support |  |  | 0.13^a^ | | 0.10^a^ |
|  | Seeking informational support |  |  |  | | -0.23^a^ |
|  | Seeking emotional support |  |  |  | | 0.05^a^ |
| *Adj. R^2^* | | 0.00 | 0.03 | | 0.06 | 0.08 |
| *R^2^* | | 0.00 | 0.03 | | 0.06 | 0.08 |
| *ΔR^2^* | | 0.00 | 0.03^a^ | | 0.03^a^ | 0.02^a^ |

*Note. All regression coefficients were standardized.*

*^a^ The association is significant at a significance level of .001 (two-tailed).*

*^b^ The association is significant at a significance level of .01 (two-tailed).*
