# Supplementary material for: Self-Disclosure and Social Support in a Web-Based Opioid Recovery Community: Machine Learning Analysis
Source: JMIR Form Res. 2025 Jul 17;9:e71207. doi: 10.2196/71207 (PMC12289226; doi:10.2196/71207)
Supplement: Multimedia Appendix 2 [file formative-v9-e71207-s002.docx]

**Appendix 2. Model Configuration and Hyperparameters**

Our study utilized eight distinct classifiers to analyze post and comment content. The "Social Support Types in comments" classifier was a multi-label classification task, as comments could simultaneously provide multiple forms of support. All other seven classifiers were binary classification tasks (e.g., Informational Self-disclosure: Yes/No) or multi-class (single-label) classification tasks (Opioid Use and Recovery Stages in posts, where a post belongs to only one stage). All these classifiers were built upon the BERT architecture, leveraging its robust capabilities for capturing complex linguistic nuances in online communities. For training these models, we consistently employed the bert-base-uncased pre-trained model.

For each classifier, a single linear layer was added on top of the base BERT model for classification. For the multi-label classification task ("Social Support Types") and all binary classification tasks, BCEWithLogitsLoss was used as the target loss function, as it is appropriate for independent binary predictions across multiple labels. For the multi-class (single-label) "Opioid Use and Recovery Stages" classifier, CrossEntropyLoss was used as the loss function, consistent with models predicting mutually exclusive categories.

Training was conducted using the Hugging Face Trainer API, configured with TrainingArguments. A consistent learning rate of 2×10^−5^ and a weight decay of 0.01 were applied using the AdamW optimizer. Training utilized a batch size of 8 for both training and evaluation. The maximum sequence length for tokenization was set to 512. Evaluation was performed after every epoch, and the F1-score was chosen as the primary metric for monitoring model performance and for selecting the best model. As a stopping criterion, models were trained for a fixed number of epochs (30 epochs for most classifiers, and 10 epochs for the Opioid Use and Recovery Stages classifier), with the best performing model based on the F1-score on the validation set being loaded at the end of training. This fixed-epoch training coupled with best-model loading serves as our stopping criterion, ensuring robust generalization. For a comprehensive breakdown of each classifier, including specific labels, maximum sequence length, and detailed hyperparameters, please refer to Table 1.

**Appendix 2. Table S7**

| **No.** | **Classifier category** | **Task type** | **BERT base model** | **Added layer -- finetuned layer** | **Loss function** | **Optimizer** | **Learning rate** | **Classes** | **Batch size** | **Epochs (fixed)** | **Weight decay** | **Metric for best model** |
| --- | --- | --- | --- | --- | --- | --- | --- | --- | --- | --- | --- | --- |
| 1 | Social Support Types (in comments) | Multi-label | bert-base-uncased | Linear Layer | BCEWithLogitsLoss | AdamW | 2.00E-05 | 6 | 8 | 30 | 0.01 | F1 |
| 2 | Opioid Use and Recovery Stages (in posts) | Multi-class | bert-base-uncased | Linear Layer | CrossEntropyLoss | AdamW | 2.00E-05 | 5 | 8 | 10 | 0.01 | F1 |
| 3 | Informational Self-disclosure (in posts) | Binary | bert-base-uncased | Linear Layer | BCEWithLogitsLoss | AdamW | 2.00E-05 | binary | 8 | 30 | 0.01 | F1 |
| 4 | Emotional Self-disclosure (in posts) | Binary | bert-base-uncased | Linear Layer | BCEWithLogitsLoss | AdamW | 2.00E-05 | binary | 8 | 30 | 0.01 | F1 |
| 5 | Seeking Emotional Support (in posts) | Binary | bert-base-uncased | Linear Layer | BCEWithLogitsLoss | AdamW | 2.00E-05 | binary | 8 | 30 | 0.01 | F1 |
| 6 | Seeking Informational Support (in posts) | Binary | bert-base-uncased | Linear Layer | BCEWithLogitsLoss | AdamW | 2.00E-05 | binary | 8 | 30 | 0.01 | F1 |
| 7 | Providing Emotional Support (in posts) | Binary | bert-base-uncased | Linear Layer | BCEWithLogitsLoss | AdamW | 2.00E-05 | binary | 8 | 30 | 0.01 | F1 |
| 8 | Providing Informational Support (in posts) | Binary | bert-base-uncased | Linear Layer | BCEWithLogitsLoss | AdamW | 2.00E-05 | binary | 8 | 30 | 0.01 | F1 |
